# A Rejoinder to "An Old Shoe's" Thoughts

**Published:** 1917-08-25

**Authors:** 


### MATRONS IN COUNCIL.

### A Rejoinder to "An Old Shoe's" Thoughts.

It is the business of the Cobbler to trace the markings of the Shoe. This old Shoe, pinching gently and without prejudice, has found obstructions in certain parts, and stops to point them out and name them. This is a great step; let us consider these definitions, and see if we are in agreement with her findings.

"A radical change is needed, but upheaval must come first." Is there not a gentler way? Is not transmutation a truer, happier method than the scrap-heap? Change the Spirit of the Dream, the outer manifestation is bound to change also.

"The Roots under the Management are sometimes old, tough, and deep." Just so, but the agriculturist knows that aeration, change of food and soil can work wonders upon roots. Change their environment, do not remove them.

Who, indeed, will roll away the Stone of Custom, the too long hours of work, the inadequate pay? Who is the strong one who can do such things? None alone has power, but strong united effort, determined effort could, an' it would; the trouble has always been, "I cannot change the Custom, I must please the management." And in pre-war days to move was extremely difficult. But if we are to learn anything from this war, surely it is that "Cannot" does not exist; "Will Not," if you like, since to accomplish means steady, undeviating pushing year in, year out, and that is work which few are strong enough to undertake; nevertheless, a determined body of all ranks, working as with one Will, speaking as with one Voice, *could* accomplish any radical change deemed necessary. Our men are not beaten abroad, nor the majority of our women at home; is the nursing profession alone to be the only one which decides to go on in the same old way, to learn no new lesson, to put forth no attempt whatever to move in line with the universal change?

### Twelve Hours on Night Duty.

Take, *e.g.*, the question of overwork. What about the long, patiently borne twelve hours of night duty? No two hours off, as in the day; all the heavy work of washing patients and making beds to be done when the worker's strength is at its lowest ebb, at the end of the long night's duty. Is this reasonable? Is it to be wondered at if they acquire the habit of tea-drinking to keep themselves going (sometimes, alas! other things), because physical need for rest is great and compelling and cannot be taken sufficiently? It is not in the three years' hospital life that these things are so harmful as far as results are concerned, for most people could stand three years' overwork if it ended there, but it is in hospital that the habit of long hours is acquired which is taken into private work and used as a basis by our women who are so faithful to the traditions of their training-schools. Why should this excessive sacrifice be demanded of any set of women? Is there any other labour in the world so heartlessly used?

### Revision!

is what the Voice cries aloud for, not upheaval. The Old Shoe says so herself further on when she alludes to the "careful loosening of old foundations." In Revision let it be remembered that the great majority of nurses go out to serve the general public as private nurses; for the safeguarding of this great majority we would plead. Their difficulties are great and not easily understood, their hours of work and rate of pay urgently need standardising, and the question of retirement and pension is very pressing.

### Wanted, a Stone Roller.

What Loving, Conscientious Soul, what Clear Brain, whatever of us is highest and most spiritual, can hope to stand up without a break against such exorbitant demands? The time must come when, through sheer lack of strength, we break or fail. What then?

Oh, Matrons, Matrons, can you not see what power lies in your hands? Use it. What matter if in the doing one or other falls by the way! Have not others done the same? And for what do we live? To do a little good, to learn a little wisdom, and then pass on.

M. C. G.

[We have the pleasure of being in a position to assure "M. C. G." that the Stone Roller and Deliverer for our nurses, whom she prays for, is in the making. A little more patience may bring harvest-time and splendid results with the uplifting of the whole profession of British nursing, to the joy of all who believe in and love it.—Ed. The Hospital.]

